# Energy Balance and Active Lifestyle: Potential Mediators of Health and Quality of Life Perception in Aging

**DOI:** 10.3390/nu11092122

**Published:** 2019-09-06

**Authors:** Giancarlo Condello, Laura Capranica, Silvia Migliaccio, Roberta Forte, Angela Di Baldassarre, Caterina Pesce

**Affiliations:** 1 Graduate Institute of Sports Training, Institute of Sports Sciences, University of Taipei, Administrative Building, 101 Zhongcheng Rd. Section 2, Shilin District, 111 Taipei, Taiwan; 2 Department of Movement, Human and Health Sciences, University of Rome Foro Italico, Piazza Lauro De Bosis 6, 00135 Roma, Italy; 3 Department of Medicine and Aging Sciences, “G. d’Annunzio” University of Chieti-Pescara, Via dei Vestini 31, 66100 Chieti, Italy

**Keywords:** physical activity, energy expenditure, body satisfaction, mental health, mediation analysis

## Abstract

The relationship between aging and perception of health and quality of life is complex and its mediation mechanisms need to be further explored. The purpose of this study was to investigate the mediating effects of total energy expenditure and intake, body mass, and body image dissatisfaction on the relationship between age and perception of health and quality of life. Forty-two senior athletes, 55 physically active, and 61 sedentary individuals (aged 55–84 years) were evaluated for total energy expenditure (EE), energy intake (EI), body mass index (BMI), absolute Body Dissatisfaction Index (BDIabx), and physical (PCS) and mental (MCS) health and quality of life perception. Multiple mediation analyses were applied to assess the relationship between age and PCS and MCS indices, through the mediators EE, EI, BMI, and BDIabx. For MCS, but not for PSC, the mediation analysis showed: (a) a direct effect of age; (b) a mediation path through EE, EI, BMI, and BDIabx; and (c) a positive total effect. The combination of positive and negative mediating effects influencing the mental health perception underlined that with advancing age, the maintenance of high levels of energy expenditure through physical activity could positively impact body image satisfaction and, in turn, mental health.

## 1. Introduction

Energy balance and active lifestyle became an imperative target for both researchers and practitioners in the modern society. Unhealthy dietary habits, inadequate level of physical activity, and extensive periods of sedentary time are the triad risk factors for major non-communicable disease [1]. They have been extensively summarized for their evidence and eminence over the life course [2]. Thus, driven by overweight pandemics, physical activity promotion has entered the agenda of supranational policies as a way to increase energy expenditure and ensure a calorie-in/calorie-out balance [3].

Considering the increase in life expectancy in developed countries, the promotion of a physically active and healthy lifestyle is of particular relevance with advancing age [4]. Aging is a multi-factorial process characterized by a combination of physical, psychological, and health factors that should be considered in light of the incidence of several chronic conditions [5]. Healthy aging has been defined as “the process of developing and maintaining the functional ability that enables well-being in older age” [6]. Therefore, it is of great concern understanding the determinants of health and quality of life in aging [7,8] and the mechanisms through which different facets of healthy aging translate into perceived health and quality of life. Indeed, the challenge for societies is not merely ensuring that the graying population ages in a healthy way, but to obtain that by aging healthily, people can also perceive a good quality of life.

Among several factors affecting aging, physical activity and diet play a key role in counteracting the decline of quality of life with advancing age [9,10,11]. Indeed, physical activity and diet synergistically contribute to energy balance, being this latter a function of body mass, energy intake, resting metabolic rate, and activity energy expenditure [12].

Advancing age causes a change in the profile of total energy expenditure and energy intake. Several studies showed a reduction in total energy expenditure in older adults compared to younger adults [13,14], due to the combined reduction in resting metabolic rate, thermogenesis, and physical activity (i.e., activity energy expenditure) [12,15]. Similarly, a concomitant reduction in energy intake can be observed [16,17]. A recent meta-analysis calculated a range of reduction in energy intake between younger (26 years of age) and older (70 years of age) individuals of about 16–20%, with an approximate decrease of 0.5% per year [18]. Moreover, the reduction in total energy expenditure reflects the concomitant decrease of body mass, related to the loss of various lean tissues, including muscle and brain [15].

A further component to be considered with advancing age is the body image, which is a multidimensional and complex construct including cognitive, perceptual, emotional, and behavioral aspects [19]. Body image could vary during the aging process, when health and physical capability become more imperative and weight control is part of the inevitable aging process. Usually, body image research is negatively framed, as it mainly focuses on body image dissatisfaction as a risk factor for a wide range of negative emotional and behavioral outcomes [20]. Body image dissatisfaction could also emerge with aging and determine negative attitudes [21], related to weight control, eating disturbances, and depression, particularly in women [20]. Even though being physically active can positively influence the body image [22], there is a lack of information about how body (dis)satisfaction interacts with further components, such as total energy expenditure, energy intake, and body mass.

The perception of health and quality of life is becoming predominant since even if a decline of physical health domain and function occurs, the overall quality of life perception can remain high. This is explained by the multifaceted nature of the perception of quality of life and the relevance of its mental health domain [7,23]. Layte and colleagues [7] found that several factors influence the quality of life, with the highest variance being explained by mental health, social participation, and physical health. Physical activity has been shown to positively impact the physical health domain and functioning, thus lowering the incidence of non-communicable diseases [24], but also emotional and cognitive components of the mental health domain [25,26]. However, aging individuals are at higher risk of being exposed to excessive physical inactivity and unhealthy eating behaviors [27]. It should be considered that physical activity is multifaceted, being defined as “any bodily movement produced by skeletal muscles that results in energy expenditure, which may be unstructured and everyday life activity, exercise that includes prearranged, deliberate and repetitive activity, and grassroots sports and competitive sports” [28]. Thus, it should be encouraged in all its forms throughout the life course, perhaps even more during aging in order to maintain adequate levels of total energy expenditure, as well physical and mental health domains and functioning. However, the constant engagement of individuals in physical activity is influenced by a series of biological, psychological, behavioral, socio-cultural, socio-economic, environmental, and policy determinants, which still deserve to be further investigated [29,30,31,32,33,34,35].

As previously proposed [36], having an active lifestyle and, in particular, being involved on competitive sport (i.e., master competitions) is a positive strategy to maintain physical and mental health domains. The three-path mediation link showed that being involved on competitive sport may contribute to maintain a healthy body mass and, in turn, to have a subjectively more satisfactory body image, therefore to positively influence the perception of mental health [36]. The abovementioned information leads to considering physical activity and its relation to body mass and image as a central component during the aging process and for the perception of health and quality of life.

However, this issue needs to be further investigated to understand whether and to what extent the energy balance issue, which dominates national and supranational policies of diet and physical activity, enters the mediation chain linking aging, physical activity, and perceived health and quality of life. Therefore, the purpose of this study was to investigate the mediating effects of total energy expenditure, energy intake, body mass, and body image dissatisfaction on the relationship between age and perception of health and quality of life.

The hypothesized mediation is based on the piecemeal evidence regarding the effects of physical activity on body mass and body image [22] and on mental health domain [25,26], the three-path mediation link between engagement in sport and mental health domain [36], and the interactive effects between BMI and quality of life [37]. The hypothesized missing link is represented by the changes in total energy expenditure and energy intake that are typically observed with advancing age.

## 2. Materials and Methods

### 2.1. Participants

The Ethics Committee Azienda Policlinico Umberto I (Rome, Italy, reference number: Prot. 451/13) approved the study and all participants provided written informed consent for participation and publication.

From an initial recruited sample of 179 individuals [36], 158 participants met the following eligibility criteria: (1) age between 55 and 84 years.; (2) not self-reported diagnosis of psychiatric or somatic illnesses; and (3) 7 complete days of evaluation of total energy expenditure and energy intake. Three types of eligible participants have been identified considering the declared physical activity level: (1) senior athletes engaged in competition (≥3 training sessions week^−1^) at national or international levels; (2) physically active individuals engaged in regular structured physical activity programs (≥2 session week^−1^); and (3) sedentary individuals engaged in ≤2 h of regular natural physical activity week^−1^. Thus, 42 were senior athletes (55−64 = 18; 65−74 = 15; 75−84 = 9), 55 physically active (55−64 = 17; 65−74 = 20; 75−84 = 18), and 61 sedentary (55−64 = 21; 65−74 = 22; 75−84 = 18).

### 2.2. Experimental Procedures

Participants reported to the laboratory in two different occasions with an interval of 10 days in between. All the evaluations took place individually under the supervision of an investigator, who specified that there were no right or wrong responses. During the first occasion, participants answered the AAHPERD (American Alliance for Health, Physical Education, Recreation, and Dance) exercise/medical history questionnaire [38] to ascertain their physical activity level, educational background, dietary habits, tobacco smoking and alcohol consumption, medication use, history of physical activity, and absence of psychiatric or somatic illnesses. Then, participants individually completed two on-line questionnaires to assess symptoms of body image dissatisfaction and perception of health and quality of life. The instruments showed Cronbach alpha coefficients ranging from 0.78 to 0.88. After the measurements of the anthropometric parameters, participants received instructions for the evaluation of the total energy expenditure and energy intake. The second occasion was necessary to ascertain the sufficient and proper data collection for the evaluation of total energy expenditure and energy intake.

### 2.3. Anthropometric Measurements

With participants wearing light underwear and no shoes, standing height, to the nearest 0.1 cm and body mass, to the nearest 0.1 kg, were measured using a portable stadiometer (Seca 220, GmbH & Co., Hamburg, Germany) and a balance scale (Seca 761, GmbH & Co., Hamburg, Germany), respectively. Body mass index (BMI, kg⋅m^−2^) was calculated to classify the participants according to the World Health Organization BMI cut-off points [39] into under-weight (<18.5 kg⋅m^−2^), normal-weight (range: 18.5–24.9 kg⋅m^−2^), overweight (range: 25.0–29.9 kg⋅m^−2^), and obese (≥30 kg⋅m^−2^) categories.

### 2.4. Steps and Total Energy Expenditure

Participants were required to wear the SenseWear Pro3 armband (BodyMedia, Pittsburgh, PA, USA). The use of SenseWear Pro armband has been already validated in older adults [40]. The armband integrates the information gathered by the two axis accelerometers and sensors (e.g., skin and near body temperature, heat flux and galvanic skin response) with sex, age, height, weight, smoking status, and handedness of the user. It provides proprietary algorithms to give quantitative information (e.g., number of daily steps, locomotor activity intensity, and total energy expenditure; [41]) about an individual’s habitual physical activity under free-living conditions involving any form of locomotion, as activities at workplace, sports, conditioning, and household. The descriptive characteristics of the participants were entered into the software program (SenseWear Professional 8; BodyMedia, Pittsburgh, PA, USA) before the monitoring was initiated. Participants wore the armband on the right arm over the triceps muscle at the midpoint between the acromion and olecranon processes. According to reliability criteria reported in the literature, participants wore the armband for more than 7 consecutive days, 24 h a day except during water-based activities [42], with a wearing time of at least 540 min⋅day^−1^ on weekdays and 480 min⋅day^−1^ on weekend days [41]. For the purpose of the present study, the mean value of the steps and total energy expenditure of 7 entire days was used for the statistical analysis.

### 2.5. Energy Intake

According to standard procedures and following previous investigations [43,44,45], participants underwent a dietary assessment. They were instructed to fill in a daily diary for a period of 7 days with the detailed food and quantity for breakfast, lunch, dinner, and snacks. They received a set of food photographs, in which three portion choices for each food item were available, in order to detect the quantity [44]. After having controlled the appropriateness of their reports, a specialized nutritionist calculated the energy intake per day considering each type of food and the relative quantity, on the base of the Italian National Institute for Research on Food and Nutrition (INRAN) [46] instructions. For the purpose of the present study, the mean value of the energy intake of 7 entire days was used for the statistical analysis.

### 2.6. Body Image Dissatisfaction

According to the standard procedures [47], individual’s body image dissatisfaction has been achieved throughout the Body Image Dimensional Assessment (BIDA). The BIDA assesses the subjective and emotional dimensions of body image by means of a neutral (i.e., not sex and not ethnic-related) silhouette-based scale. Participants had to indicate their perceived and ideal body shape, the most appropriate body shape for their peers, and the most appreciated body shape by the opposite sex. They were not limited to selecting numerical values corresponding to images appearing on the scale, but they could indicate intermediate values using a scale ranging from 1.8 to 5.2 for which there are no representative images. Thus, body dissatisfaction (BD), sexual body dissatisfaction (SxBD), comparative body dissatisfaction (CBD), and body dissatisfaction index (BDI) in relation to body size were calculated, with absolute body dissatisfaction index (BDIabx) > 30% being considered at risk of body image disorders.

### 2.7. Health and Quality of Life Perception

Following the standard procedures [48] and previous investigations [36,49,50], the perception of health and quality of life has been evaluated using the Short Form Health Survey Version 2^®^ (SF-12v2). The instrument consists of 12 questions covering eight health domains: (1) physical functioning; (2) role limitations due to physical problems; (3) bodily pain; (4) general health perception; (5) energy and vitality; (6) social functioning; (7) role limitations due to emotional problems; and (8) mental health. The eight scores were aggregated into two summary measures representing the perception of health and quality of life in the physical (physical component summary, PCS) and mental (mental component summary, MCS) health domains. The two scores could range from 0 (i.e., lowest level of health) to 100 (i.e., highest level of health), with the cut-point for considering below or above the mean value of the general population norm set at 50 ± 10 pt. [49].

### 2.8. Statistical Analysis

Data were analyzed using the Statistical Package for the Social Science, version 25.0 (SPSS Inc., Chicago, IL, USA). The level of statistical significance was set at *p* < 0.05 for all computations. Prior to the analysis, the Kolmogorov–Smirnov test was applied to ascertain the normality of data distribution for each group.

Initially, a preliminary inferential analysis was applied to the sample. A 3 × 3 × 2 multivariate analysis of variance (MANOVA) was applied to ascertain the effect of physical activity level (senior athletes, physically active, sedentary), age class (55–64, 65–74, 75–84 years), and gender on the variables studied: anthropometric characteristics, BMI, number of medications and diseases; steps, total energy expenditure, and energy intake; body image dissatisfaction (BD, SxBD, CBD, BDIabx); perception of health and quality of life in the physical and mental health domains (PCS, MCS). Planned pairwise *t*-tests with Bonferroni correction were used for multiple post-hoc comparisons. Effects sizes were calculated as partial eta squared (*η*_*ρ*_^2^) for ANOVA results.

In accordance to the preliminary analysis and to the related findings on the perception of health and quality of life in the physical and mental health domains (PCS, MCS), a mediation analysis was applied to assess whether the expected relationship between age and perception of health and quality of life was explained by mechanisms that involved total energy expenditure, energy intake, body mass, and body image dissatisfaction. Using the SPPS macro PROCESS, two serial multiple mediation analyses (one for PCS and one for MCS) were performed for the evaluation of the following effects: (1) the independent variable (X: age) on the dependent variable (Y: PCS or MCS); (2) the independent variable (X) on each mediator (M: total energy expenditure, energy intake, BMI, BDIabx); (3) the independent variable (X) and the potential mediators (M) on the dependent variable (Y). Bootstrapping was applied to empirically estimate the sampling distribution of the indirect effect and generate a bootstrap confidence interval (95% CI) based on 10,000 bootstrap samples for bias corrected bootstrap CIs. This CI was used as a form of hypothesis test to estimate if the size of the indirect effect of each individual mediator was different from zero [51].

## 3. Results

Anthropometric characteristics, BMI, regular use of medications, and number of diseases of the participants are reported in Table 1. Main effects were found for physical activity level in respect to BMI (*F*_(2,155)_ = 4.671, *p* = 0.011, *η*_*ρ*_^2^ = 0.063) and medications (*F*_(2,155)_ = 10.291, *p* < 0.001, *η*_*ρ*_^2^ = 0.128), for age class in respect to height (*F*_(2,155)_ = 14.250, *p* < 0.001, *η*_*ρ*_^2^ = 0.169), body mass (*F*_(2,155)_ = 4.934, *p* = 0.009, *η*_*ρ*_^2^ = 0.066), medications (*F*_(2,155)_ = 3.447, *p* = 0.035, *η*_*ρ*_^2^ = 0.047), diseases (*F*_(2,155)_ = 7.666, *p* = 0.001, *η*_*ρ*_^2^ = 0.099), and for gender in respect to height (*F*_(1,156)_ = 116.310, *p* < 0.001, *η*_*ρ*_^2^ = 0.454) and body mass (*F*_(1,156)_ = 58.116, *p* < 0.001, *η*_*ρ*_^2^ = 0.293). Regarding the physical activity level effect, post hoc analysis maintained differences in BMI (*p* = 0.008) between senior athletes and sedentary counterparts, and in medications for senior athletes with respect to both physically active (*p* < 0.001) and sedentary (*p* < 0.001) counterparts. Regarding the age class effect, post hoc analysis maintained differences in height for 55–64 years-old individuals with respect to 65–74 (*p* < 0.001) and 75–84 (*p* < 0.001) counterparts, in body mass between 55–64 and 75–84 year-old individuals (*p* = 0.010), and in diseases for 55–64 year-old individuals with respect to 65–74 (*p* = 0.006) and 75–84 (*p* = 0.003) counterparts.

### 3.1. Preliminary Analysis

The preliminary analysis demonstrated main effects for physical activity level (*F*_(2,155)_ = 6.551, *p* = 0.002, *η*_*ρ*_^2^ = 0.086), age class (*F*_(2,155)_ = 20.460, *p* < 0.001, *η*_*ρ*_^2^ = 0.226), and gender (*F*_(1,156)_ = 69.906, *p* < 0.001, *η*_*ρ*_^2^ = 0.333) in respect to total energy expenditure, whilst only a gender main effect for energy intake (*F*_(1,156)_ = 29.993, *p* < 0.001, *η*_*ρ*_^2^ = 0.176). In consideration of steps, a main effect for physical activity level (*F*_(2,155)_ = 5.652, *p* = 0.004, *η*_*ρ*_^2^ = 0.075) and age class (*F*_(2,155)_ = 5.963, *p* = 0.003, *η*_*ρ*_^2^ = 0.078) emerged. Regarding the physical activity level effect, post hoc analysis maintained differences for sedentary individuals with respect to senior athletes and physically active counterparts, for both total energy expenditure (*p* = 0.002 and *p* = 0.024, respectively) and steps (*p* = 0.034 and *p* = 0.011, respectively). Regarding age class effect, post hoc analysis confirmed differences among the three age classes (55–64 vs. 65–74, *p* < 0.001; 55–64 vs. 75–84, *p* < 0.001; 65–74 vs. 75–84, *p* = 0.012) for total energy expenditure, whilst differences were confirmed in steps for 75–84 year-old individuals with respect to 55–64 (*p* = 0.004) and 65–74 (*p* = 0.007) counterparts. Regarding gender effect, for both total energy expenditure and energy intake, male individuals showed higher values (*p* < 0.001) compared to female counterparts (Table 2).

Considering body image dissatisfaction, a main effect was found for physical activity level in respect to BD (*F*_(2,155)_ = 3.405, *p* = 0.036, *η*_*ρ*_^2^ = 0.046), SxBD (*F*_(2,155)_ = 4.704, *p* = 0.011, *η*_*ρ*_^2^ = 0.063), CBD (*F*_(2,155)_ = 6.877, *p* = 0.001, *η*_*ρ*_^2^ = 0.089), and BDIabx (*F*_(2,155)_ = 3.177, *p* = 0.045, *η*_*ρ*_^2^ = 0.043), and for age class in respect to CBD (*F*_(2,155)_ = 3.626, *p* = 0.029, *η*_*ρ*_^2^ = 0.049). Post hoc analysis showed that senior athletes differed from the sedentary counterpart in BD (*p* = 0.034), SxBD (*p* = 0.016), and CBD (*p* = 0.002), while physically active individuals differed from sedentary counterpart in BDIabx (*p* = 0.039). For the age class effect on CBD, only 55–64 year-old individuals significantly differed (*p* = 0.04) from 75–84 counterpart.

Regarding health and quality of life perception in the physical and mental health domains, a main effect emerged for physical activity level with respect to MSC only (*F*_(2,155)_ = 3.371, *p* = 0.037, *η*_*ρ*_^2^ = 0.046). However, this effect was not confirmed in the post hoc analysis, with the difference between senior athletes and sedentary counterpart only approaching the significance (*p* = 0.053). Furthermore, a main effect emerged for age class (*F*_(2,155)_ = 4.489, *p* = 0.013, *η*_*ρ*_^2^ = 0.060). The post hoc analysis maintained a significant difference between 55–64 and 75–84 year-old individuals (*p* = 0.025) with the younger age class reporting a lower perception in mental health domain compared to the older age class (Table 3).

### 3.2. Mediation Analysis

**Figure 1:**
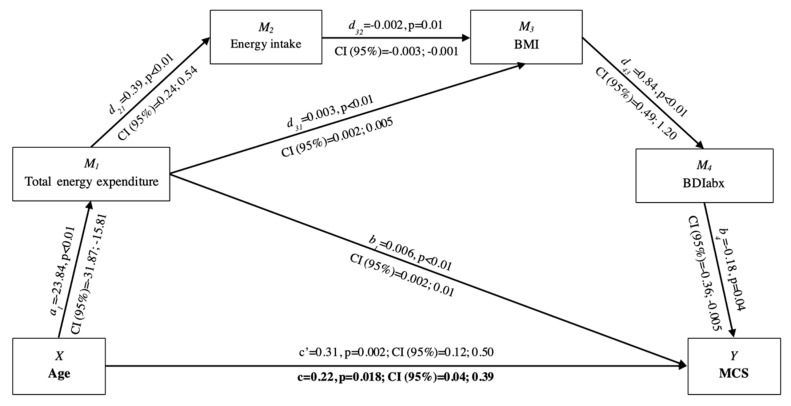
Conceptual and statistical model of age and mediators’ effects on MCS. c’ = direct effect; c = total effect. BMI = body mass index. BDIabx (absolute body dissatisfaction index), MCS = mental component summary.

The serial mediation analysis showed the existence of significant paths between independent, dependent, and mediating variables only in the case of MCS (Figure 1). Firstly, there was a direct positive effect (c’) of age on MCS. Secondly, the relationship was mediated by the four mediators. As indicated by the bootstrapping output, a serial indirect effect path existed, including total energy expenditure, energy intake, BMI, and BDIabx, as the 95% CI of bootstrap estimates of the indirect effect did not include the zero value (−0.003, Bootstrap 95% CI = −0.011; −0.0002). However, the analysis indicated also the presence of other significant serial indirect effect paths. The relationship between age and MCS was mediated by the total energy expenditure alone (−0.135, Bootstrap 95% CI = −0.251; −0.048), and by total energy expenditure, BMI, and BDIabx jointly (−0.0113, Bootstrap 95% CI = −0.001; −0.038). Taken together, all the indirect effects provided a negative total indirect effect (−0.094, Bootstrap 95% CI = −0.203; −0.001). Although this mediation analysis represented an example of inconsistent mediation, since the mediating effects had a different sign, the resulting total effect (c) had a positive value (Figure 1).

## 4. Discussion

The main purpose of this study was to investigate the mediating effects of total energy expenditure, energy intake, body mass, and body image dissatisfaction on the relationship between age and perception of health and quality of life. The mediation analysis showed a series of effects building a mediation path only in the case of health and quality of life perception in the mental health domain (from here on: mental health perception). The application of mediation analysis is confirmed to be an appropriate methodology for exploring interactive effects among several variables and informing policy-makers and implementation professionals, as already suggested in previous research in older adults [11,36]. The novelty of this study is the linked role played by energy balance and body mass and body image dissatisfaction in the relationship between age and mental health perception. Figure 1 shows how this relationship was mediated by a serial indirect effect path encompassing total energy expenditure, energy intake, body mass, and body image dissatisfaction. Whilst a previous mediation analysis showed that the mental health perception could be mediated by body mass and body image dissatisfaction [36], this study adds novel elements to the explanatory mediation chain, linking advancing age with energy balance, body mass, and body image dissatisfaction for the understanding of the age-related changes in the perception of health and quality of life.

Energy balance is a function of body mass, energy intake, resting metabolic rate, and activity energy expenditure [12]. The preliminary analysis regarding the effect of physical activity level, age class, and gender confirmed previous investigations regarding the differences between male and female individuals in terms of total energy expenditure and energy intake [17,52,53,54]. Furthermore, total energy expenditure decreased together with number of steps per day, with the sedentary and older individuals showing significantly lower values compared to more active and younger counterparts. Therefore, it is always important to promote the maintenance of high levels of physical activity during aging to reduce the risk of chronic diseases [24]. Moreover, mental health perception was significantly different between the youngest and oldest age class. Therefore, the mediation analysis has been built to investigate the relationship between age and the perception of health and quality of life, adding the influence of energy balance components, which were not included in a previous mediation analysis [36].

The main analysis of the present study showed that neither age nor the four mediators had direct and/or indirect effects on the health and quality of life perception in the physical health domain (from here on: physical health perception). This finding supports a previous investigation showing that physical health perception is not the principal component when investigating perceived health. In fact, the mental health (7.7%) and social participation (6.3%) factors explained a higher variance of the variation of quality of life than physical health (3.7%) [7]. The PCS of the SF-12v2 includes the best two scales to predict physical health, such as physical functioning and physical role. The mean PCS values of the overall sample (51.8 ± 7.4 pt.) and for each factor (i.e., physical activity level, age class, and gender; Table 3) were slightly above the mean of the general population norm (50 ± 10 pt.) [49]. Therefore, the sample of the present study showed a satisfactory physical health perception without impacting the overall health and quality of life perception.

Conversely, a direct and positive effect of age on mental health perception emerged. Thus, with advancing age there seems to be, counter-intuitively, a better perception of mental health. Indeed, the mean MCS value of the overall sample (51.4 ± 9.1 pt.) was slightly above the mean of the general population norm (50 ± 10 pt.) [49], whilst considering each factor (i.e., physical activity level, age class, and gender; Table 3), sedentary, 55–64 yrs., and female subgroups were slightly below the mean of the general population norm (50 ± 10 pt.) [49]. The direct effect found from the mediation analysis reinforces the view that life satisfaction and health perception is not entirely influenced by activities of daily living capacity, as demonstrated in six European countries [23]. Similarly, Thomas and colleagues [55] found a better mental health in the older cohorts compared to younger cohorts, even despite a worsening of physical and cognitive function. As reported by the authors, several theories have been proposed in the literature to explain this “paradoxical trend”, as favorable brain changes, reduced mental distress, better capability to cope with stressful changes, socioemotional selectivity in later life, increase in wisdom, better ability for emotional regulation and complex social decision making [55].

This mediation analysis represented an example of inconsistent mediation, since the total indirect effect (as well as the serial indirect effect of the four mediators) had a negative sign, and thus an opposite effect compared to the direct effect (i.e., c’). It has been previously reported that a higher possibility of inconsistent mediation could occur when there is a higher number of mediators [56]. This reason would explain the findings of the present study. Analyzing the serial indirect effect path, advancing age was associated with a decrease in total energy expenditure, a reduction in energy intake, an increase in BMI, which caused a higher body image dissatisfaction, and thus a lower mental health perception. Except for the relationship between energy intake and BMI, all the other relationships appeared as expected and they were in line with previous studies [12,13,14,15,16,17,18]. Conversely, differently from the expectations, for a lower energy intake it would correspond a higher BMI. However, previous studies investigating the relationship between these two variables showed contrasting results. Trichopoulou and colleagues [57] reported a positive association between energy intake and BMI, even though the magnitude of correlation was weak. Opposite results emerged from other studies and one explanation has been attributed to the underreport of dietary intake [58,59]. Furthermore, the relationship between energy intake and BMI could depend on the size of BMI, being stronger only for higher values of BMI (i.e., >27.2 kg⋅m^−2^ for males and >23.8 kg⋅m^−2^ for females, respectively) [60]. Further explanations could be diet quality (i.e., scarce diet quality is associated with higher BMI) [61] and the frequency of meals (i.e., negative association between frequency and BMI) [62].

Aging remains a multifaceted process involving several factors and during which different changes could have a positive and/or negative impact on the physical and mental health domains of perception of health and quality of life. However, as also demonstrated by the mediation analysis, it seems total energy expenditure played a predominant role and drove other mediators. A decrease or increase in total energy expenditure guides the other mediators towards a body (dis)satisfaction and, in turn, a lower or higher mental health perception. Moreover, total energy expenditure is directly linked to the amount of physical activity. Thus, the maintenance of a high level of physical activity remains always the first strategy to counteract aging-related declines. In fact, the findings of the present study strengthen the recent World Health Organization global action plan [3], which further emphasized the promotion of physical activity across life course. Regional and national coordination and global leadership are required to integrate their efforts in supporting all people to be regularly active. Policy-makers and stakeholders should cooperate in order to provide efficient solutions for health-enhancing physical activity integrating several sectors (i.e., health, sports, transport, urban design, civil society, academia, and the private sector) and considering the interaction between all the determinants of physical activity behavior at personal and society level [28].

The present study has some limitations which need to be addressed and could serve as a guidance for future research. Firstly, the present study adopted a cross-sectional design. Future research should be conducted with longitudinal and intervention study designs maintaining the mediation analysis in order to evaluate the mediating effects on the relationship between age and perception of health and quality of life. Secondly, even though the dietary diary has been extensively used to estimate energy intake, it maintains the limitations of being time consuming for the participants and it could produce an underestimation of the total energy intake. However, in the present study, the choice of dietary assessment for 7 days has been taken to counteract the possible effect of day-to-day variations in energy intake, even if it required more time and effort. Furthermore, it was not possible to evaluate the contribution of single macronutrients (i.e., carbohydrate, protein, fat) to energy intake. Thus, future studies could be addressed at evaluating the macronutrients to provide further diet-related implications for older adults in counteracting sarcopenia. Last, in the present study, only BMI has been calculated, without a clear understanding of the contribution of both muscle and fat mass. Considering the contrasting results regarding energy intake and BMI, future studies could further explore and clarify their relationship adopting randomized controlled trails and using a different method for the evaluation of body composition, in order to evaluate the cause–effect relationships.

## 5. Conclusions

The present study confirmed the importance of energy balance with advancing age because of its linkage to body mass and body image dissatisfaction, which, in turn, affect the perception of health and quality of life in aging individuals. Since the major source of energy expenditure is physical activity, the present results support the relevance of lifelong participation in exercise and sport for healthy aging and maintenance of quality of life, also and in particular in the mental health domain. Indeed, the maintenance of elevated levels of physical activity plays a crucial role since it can influence the initial and primary mediator of the path, such as the total energy expenditure.

In sum, the understanding of the mediational mechanisms of energy balance, body mass, and body image dissatisfaction can help to develop appropriate strategies to maintain a good perception of health and quality of life during later life, as the reduction of mental distress, a better capability to cope with stressful changes, socioemotional selectivity, and better emotional regulation.

## Figures and Tables

**Table 1:** Anthropometric characteristics, BMI, number of medications, and diseases (mean ± SD)

	Body Mass (kg)	Height (m)	BMI (kg·m^−2^)	Medications (n)	Diseases (n)
**Physical activity level**					
Senior athletes	69.4 ± 10.2	1.67 ± 0.07	24.80 ± 2.75 ^1^	1.3 ± 1.2 ^1,2^	1.8 ± 2
Physically active	74.0 ± 12.3	1.65 ± 0.09	27.19 ± 3.77	3.7 ± 2.9	2.9 ± 2.3
Sedentary	76.0 ± 14.6	1.64 ± 0.1	28.10 ± 3.73	3.7 ± 2.9	3.1 ± 2.9
**Age class**					
55–65	76.6 ± 14.5 ^3^	1.68 ± 0.09 ^3,4^	26.95 ± 4.2	2.1 ± 2	1.5 ± 2.2 ^3,4^
65–74	73.7 ± 12.8	1.64 ± 0.09	27.37 ± 3.61	3.2 ± 2.6	2.9 ± 2.3
75–84	69.6 ± 9.9	1.63 ± 0.09	26.26 ± 3.26	4.0 ± 3.3	3.8 ± 2.6
**Gender**					
Female	65.5 ± 9.8	1.59 ± 0.06	26.08 ± 3.98	3.3 ± 3.3	2.8 ± 2.5
Male	80.0 ± 11.5 ^5^	1.70 ± 0.07 ^5^	27.56 ± 3.42	2.9 ± 2.3	2.6 ± 2.5

BMI = body mass index. ^1^ Significant difference from sedentary counterpart. ^2^ Significant difference from physically active counterpart. ^3^ Significant difference from 75–84 counterpart. ^4^ Significant difference from 65–74 counterpart. ^5^ Significant difference from female counterpart.

**Table 2:** Steps, total energy expenditure, and energy intake in relation to physical activity level, age class, and gender (mean ± SD).

	Steps (n)	Total Energy Expenditure (Kcal·day^−1^)	Energy Intake (Kcal·day^−1^)
**Physical activity level**			
Senior athletes	11,941 ± 3739 ^1^	2553.3 ± 468.4 ^1^	1961.7 ± 368.1
Physical active	10,819 ± 3601 ^1^	2483.5 ± 460 ^1^	1884.4 ± 353.4
Sedentary	8923 ± 3218	2318.7 ± 423.9	1806.3 ± 489.3
**Age class**			
55–65	11,1834 ± 3495 ^2^	2653.9 ± 492.2 ^2,3^	1926.7 ± 384.1
65–74	10,785 ± 3919 ^2^	2402.4 ± 370.9 ^2^	1818.3 ± 390.3
75–84	8884 ± 3230	2215.9 ± 393.5	1881.8 ± 483.6
**Gender**			
Female	10,915 ± 3747	2178.4 ± 337	1675.7 ± 342
Male	9964 ± 3609	2645.2 ± 434.4 ^4^	2033.2 ± 404.3 ^4^

^1^ Significant difference from sedentary counterpart. ^2^ Significant difference from 75–84 counterpart. ^3^ Significant difference from 65–74 counterpart. ^4^ Significant difference from female counterpart.

**Table 3:** BIDA and SF-12v2 indexes (pt) in relation to physical activity level, age class, and gender (mean ± SD)

	BD (%)	SxBD (%)	CBD (%)	BDIabx (%)	PCS (pt.)	MCS (pt.)
**Physical activity level**						
Senior athletes	9.4 ± 13.9 ^1^	4.8 ± 23.7 ^1^	−18.3 ± 18.9 ^1^	17.9 ± 8.6	54.5 ± 5.1	53.6 ± 7.7
Physically active	14.9 ± 11.9	12.1 ± 17.5	−11.8 ± 16.0	15.8 ± 7.2 ^1^	51.8 ± 7.3	51.9 ± 8.7
Sedentary	18.7 ± 13.9	20.0 ± 18.4	−2.7 ± 22.4	19.7 ± 9.7	50.0 ± 8.3	49.5 ± 10.1
**Age class**						
55–65	17.8 ± 13.1	17.2 ± 18.4	−4.8 ± 21.0 ^2^	18.9 ± 9.7	52.6 ± 6.8	48.7 ± 10.7 ^2^
65–74	14.7 ± 14.3	14.5 ± 21.2	−10.9 ± 21.9	18.7 ± 8.1	50.4 ± 8.1	52.0 ± 7.8
75–84	11.6 ± 13.1	6.5 ± 20.9	−15.4 ± 15.8	15.5 ± 7.9	52.6 ± 7.0	53.9 ± 7.8
**Gender**						
Female	16.3 ± 14.1	13.0 ± 19.6	−8.9 ± 20.5	17.6 ± 9.4	51.1 ± 7.7	49.6 ± 10.6
Male	13.7 ± 13.3	13.3 ± 21.2	10.8 ± 20.3	18.1 ± 8.2	52.4 ± 7.2	52.8 ± 7.5

BD = body dissatisfaction, SxBD = sexual body dissatisfaction. CBD = comparative body dissatisfaction, BDIabx (absolute body dissatisfaction index), PCS = physical component summary, MCS = mental component summary.^1^ Significant difference from sedentary counterpart. ^2^ Significant difference from 75–84 counterpart.
